# Changes in Free Amino Acid Concentration in Rye Grain in Response to Nitrogen and Sulfur Availability, and Expression Analysis of Genes Involved in Asparagine Metabolism

**DOI:** 10.3389/fpls.2016.00917

**Published:** 2016-06-22

**Authors:** Jennifer Postles, Tanya Y. Curtis, Stephen J. Powers, J. S. Elmore, Donald S. Mottram, Nigel G. Halford

**Affiliations:** ^1^Plant Biology and Crop Science Department, Rothamsted ResearchHarpenden, UK; ^2^Biotechnology and Biological Sciences Research CouncilSwindon, UK; ^3^Computational and Systems Biology Department, Rothamsted ResearchHarpenden, UK; ^4^Department of Food and Nutritional Sciences, University of ReadingReading, UK

**Keywords:** acrylamide, food safety, asparagine synthetase, glutamine synthetase, asparaginase, aspartate kinase, GCN2, SnRK1

## Abstract

Free asparagine plays a central role in nitrogen storage and transport in many plant species due to its relatively high ratio of nitrogen to carbon. However, it is also a precursor for acrylamide, a Class 2a carcinogen that forms during high-temperature processing and cooking. The concentration of free asparagine was shown to increase by approximately 70% in rye grain in response to severe sulfur deficiency (*F*-test, *p* = 0.004), while the concentration of both free asparagine and free glutamine increased (by almost threefold and approximately 62%, respectively) in response to nitrogen application (*F*-test, *p* < 0.001 for free asparagine; *p* = 0.004 for free glutamine). There were also effects of nutrient supply on other free amino acids: The concentration of free proline, for example, showed a significant (*F*-test, *p* = 0.019) effect of nitrogen interacting with sulfur, with the highest concentration occurring when the plants were deprived of both nitrogen and sulfur. Polymerase chain reaction products for several genes involved in asparagine metabolism and its regulation were amplified from rye grain cDNA. These genes were asparagine synthetase-1 (*ScASN1*), glutamine synthetase-1 (*ScGS1*), potassium-dependent asparaginase (*ScASP*), aspartate kinase (*ScASK*), and general control non-derepressible-2 (*ScGCN2*). The expression of these genes and of a previously described sucrose non-fermenting-1-related protein kinase-1 gene (*ScSnRK1*) was analyzed in flag leaf and developing grain in response to nitrogen and sulfur supply, revealing a significant (*F*-test, *p* < 0.05) effect of nitrogen supply on *ScGS1* expression in the grain at 21 days post-anthesis. There was also evidence of an effect of sulfur deficiency on *ScASN1* gene expression. However, although this effect was large (almost 10-fold) it was only marginally statistically significant (*F*-test, 0.05 < *p* < 0.10). The study reinforced the conclusion that nutrient availability can have a profound impact on the concentrations of different free amino acids, something that is often overlooked by plant physiologists but which has important implications for flavor, color, and aroma development during cooking and processing, as well as the production of undesirable contaminants such as acrylamide.

## Introduction

Free asparagine plays a central role in nitrogen storage and transport in many plant species due to its relatively high ratio of nitrogen to carbon (Lea et al., 2007). However, interest in its metabolism and accumulation has greatly increased recently due to its role in the formation of acrylamide (C_3_H_5_NO), a processing contaminant that is produced in the Maillard reaction, a series of non-enzymic reactions between reducing sugars such as glucose, fructose and maltose, and free amino acids (Halford et al., 2011). The Maillard reaction occurs at the high temperatures generated by frying, baking, roasting, or high-temperature processing, and is also responsible for the production of melanoidin pigments and complex mixtures of compounds that impart the flavors and aromas that are associated with fried, baked, and roasted foods. Acrylamide forms principally *via* the deamination and decarboxylation of asparagine (Mottram et al., 2002; Stadler et al., 2002). In wheat, rye and almost certainly other cereals, free asparagine concentration is the determining factor for acrylamide-forming potential (reviewed by Curtis et al., 2014).

Acrylamide is classified as a Group 2A carcinogen and the latest report from the European Food Safety Authority (EFSA)’s Expert Panel on Contaminants in the Food Chain (CONTAM) stated that the margins of exposure for acrylamide indicate a concern for neoplastic effects based on animal evidence (EFSA Panel on Contaminants in the Food Chain (CONTAM), 2015). The European Commission issued ‘indicative’ levels for the presence of acrylamide in food in 2011 and revised them downward for many products in 2013 (European Commission, 2013). Furthermore, in response to the CONTAM report the Commission is currently reviewing additional options for risk management measures.

Cereal products are major contributors to dietary acrylamide intake in Europe, accounting in adults for between 32% of intake in the UK and 45% in Germany (European Food Safety Authority, 2011). Bread alone accounts for 15% of intake in the UK, 25% in France and 32% in Germany. The food industry has devised many strategies for reducing acrylamide formation by modifying food processing and these have been compiled in a ‘Toolbox’ produced by Food Drink Europe (2013). However, these methods do not work for all products, and producing cereal grains with lower and more predictable acrylamide-forming potential would help the food industry to comply with regulations without costly changes to manufacturing processes.

Free asparagine accumulates to high concentrations during processes such as seed germination and in response to a range of abiotic and biotic stresses (Forde and Lea, 2007; Lea and Azevedo, 2007; Lea et al., 2007; Halford et al., 2015). For example, it accumulates in *Hordeum* species in response to salt stress (Garthwaite et al., 2005), together with proline and glycine betaine (an N-trimethylated amino acid), while there is a 15- and 28-fold rise in the concentration of free asparagine and proline, respectively, in pearl millet (*Pennisetum glaucum*) in response to drought stress (Kusaka et al., 2005). Indeed, free asparagine becomes the predominant free amino acid in cereal grains under some stress conditions, and there is evidence from several studies that its concentration varies considerably in the grain of both wheat (*Triticum aestivum*) and rye (*Secale cereale*) sourced from different locations or grown in different years, showing that its metabolism is responsive to multiple environmental and crop management factors (Taeymans et al., 2004; Baker et al., 2006; Claus et al., 2006; Curtis et al., 2009, 2010; Halford et al., 2012). The fact that free asparagine and other free amino acids accumulate to high concentrations in plant tissues in response to stress is an example of how stress can have profound effects on crop composition as well as yield (Halford et al., 2015).

Free asparagine concentration has been shown to correlate positively with nitrogen availability in the grain of barley (*Hordeum vulgare*) (Winkler and Schön, 1980), wheat (Martinek et al., 2009), and rye (Postles et al., 2013), while deficiencies in other minerals become important when there is a plentiful supply of nitrogen (reviewed by Lea et al., 2007). Sulfur deficiency in particular can cause a massive (up to 30-fold) increase in free asparagine accumulation in wheat, barley, and maize (*Zea mays*) (Shewry et al., 1983; Baudet et al., 1986; Muttucumaru et al., 2006; Granvogl et al., 2007; Curtis et al., 2009), although in rye the response is much less dramatic, at least under field conditions (Postles et al., 2013). Consistent with this, asparagine synthetase gene expression has been shown to increase in wheat seedlings and, to a lesser extent, grain under sulfur-limited growth conditions (Byrne et al., 2012; Gao et al., 2016). The aim of the present study was to undertake a glasshouse-based investigation of the effect of nitrogen and sulfur availability on free amino acid accumulation in rye grain, and to investigate changes in expression of genes that could be associated with free asparagine concentration. By conducting the study in a glasshouse it was possible to impose a more severe degree of sulfur deficiency on the plants than would be possible in the field.

The genes that were chosen for study were asparagine synthetase-1 (*ASN1*), glutamine synthetase-1 (*GS1*), potassium-dependent asparaginase (*ASP*), aspartate kinase (*ASK*), general control non-derepressible-2 (*GCN2*), and sucrose non-fermenting-1-related protein kinase-1 (*SnRK1*). Asparagine synthetase catalyses the formation of asparagine *via* the amidation of aspartate, with glutamine donating an ammonium group. Four asparagine synthetase genes have been identified in wheat (*TaASN1-4*; Gao et al., 2016), maize (Todd et al., 2008; Duff et al., 2011) and barley (Avila-Ospina et al., 2015), and they show differential tissue-specific and developmental regulation of expression. In wheat, *TaASN2* is the gene most highly expressed in the grain, but *TaASN1* is the gene most responsive to sulfur availability (Byrne et al., 2012; Gao et al., 2016), hence the choice of *ScASN1* for this study in rye. The control of *TaASN1* gene expression in response to sulfur availability appears to involve a GCN2-type protein kinase (Byrne et al., 2012), and a *GCN2* gene was also included in the study.

SnRK1 is another protein kinase implicated in the control of asparagine synthetase gene expression, at least in *Arabidopsis* (*Arabidopsis thaliana*). SnRK1 has a range of roles in sugar and stress signaling (reviewed by Hey et al., 2010) and reporter gene expression driven by the *Arabidopsis ASN1* gene promoter has been shown to be greatly increased by SnRK1 over-expression (Baena-Gonzalez et al., 2007; Baena-González and Sheen, 2008; Confraria et al., 2013). SnRK1 was first discovered in rye (originally called RKIN1; Alderson et al., 1991) and analysis of expression of rye *SnRK1* was also included in the study.

GS1 is the cytosolic form of the enzyme that catalyses the ATP-dependent condensation of glutamate and ammonia to form glutamine (Bernard and Habash, 2009). It is responsible for the assimilation of ammonium following nitrate reduction, as well as the production of glutamine for the transport of nitrogen between organs and the recycling of nitrogen released during catabolic processes. Plants also contain a plastidic form of the enzyme, GS2 (Lam et al., 1996), but this enzyme is involved in the recapturing of nitrogen lost as NH_4_^+^ through photorespiration and was therefore considered less relevant to the study.

Asparaginase catalyses the hydrolysis of the amide group of asparagine to release aspartate and ammonia, the latter being reincorporated into amino acid metabolism by glutamine synthetase, while monofunctional aspartate kinase (ASK) catalyses the phosphorylation of aspartate (the first step in the biosynthesis of the ‘aspartate family’ amino acids: methionine, lysine, and threonine) and therefore potentially competes with asparagine synthetase for aspartate.

## Materials and Methods

### Growth of Rye Plants with Different Combinations of Nitrogen and Sulfur Feeding

Seedlings of three rye (*Secale cereale*) varieties, Agronom, Askari, and Festus, were vernalised for 8 weeks then transferred to 21 cm pots, with five plants per pot, in a glasshouse with a 16 h day length. The pots contained vermiculite [general formula: (Na_0.21_, K_0.39_, Mg_0.19_, Ca_0.13_, 6H_2_O) (Mg_5_, Fe^+2^_0.2_, Fe^+3^_0.8_) [Si_5.5_, Al_2.5_, O_20_] (OH)_4_] and the plants were therefore reliant on supplied minerals. These were provided in a medium containing a full nutrient complement of potassium, phosphate, calcium, magnesium, sodium, and iron, plus combinations of nitrate at a full rate (N^+^) of 2 mM Ca(NO_3_)_2_ and 1.6 mM Mg(NO_3_)_2_ or a deficient rate (N^-^) at 5% of the full rate, together with sulfate at a full rate (S^+^) (1.1 mM MgSO_4_) or deficient rate (S^-^) with no added sulfate (Muttucumaru et al., 2006; Curtis et al., 2009; Byrne et al., 2012). The four nitrogen × sulfur combinations (N^+^S^+^; N^+^S^-^; N^-^S^+^; N^-^S^-^) together with the three varieties gave a total of 12 variety × treatment combinations. These were arranged in a randomized block design in three blocks, the three blocks providing biological replicates. Feeding was started 3 weeks after potting and continued every 2 days until harvest. The five plants per pot allowed destructive sampling of an individual plant in each pot at a pre-anthesis time point and then at 7, 14, 21, and 28 days post anthesis (dpa). Distilled water was supplied when necessary to prevent water stress.

### Measurement and Statistical Analysis of Free Amino Acid Concentrations

Amino acid concentrations in wholemeal flour at 21 and 28 dpa were measured by extraction in 0.01 N HCl, derivatisation using the EZ: Faast free amino acid kit (Phenomenex, Torrance, CA, USA), and analysis by gas chromatography-mass spectrometry (GC-MS) using an Agilent 6890 GC-5975-MS system (Agilent, Santa Clara, CA, USA) in electron impact mode. The method has been described previously by Postles et al. (2013) and was based on that of Elmore et al. (2005). Initial analyses of the data were performed using the Agilent Chemstation data analysis software. The GenStat^®^ statistical software (2010, Thirteenth Edition, VSN International Ltd, Hemel Hempstead, UK) was then used to test (*F*-tests) the main effects of and interactions between variety, S, N, and development time factors on the concentrations of free amino acids, using analysis of variance (ANOVA). A natural log (to base *e*) transformation was applied to account for some heterogeneity of variance over the variety × S × N × development time combinations. The least significant difference (LSD) at the 5% (*p* = 0.05) level, calculated from the standard error of the difference (SED) between means on the residual degrees of freedom (df) from the ANOVA, was used to make comparison of relevant means.

### RNA Extraction

For cDNA synthesis and cloning, frozen rye cv. Askari seedlings were ground to a fine powder with a chilled pestle and mortar, using liquid nitrogen to keep the material frozen. Total RNA extraction from 0.1 g of the powdered tissue was performed using the RNeasy Mini Kit (Qiagen Ltd, Crawley, UK), according to the protocol provided by the manufacturer. RNA quality was assessed by electrophoresis on an agarose gel and the quantity of total RNA was measured using a NanoDrop spectrophotometer (Thermo Fisher, Wilmington, DE, USA; supplied by Labtech International Ltd, Uckfield, UK). DNA contamination was removed by incubation with DNaseI at 37°C (Promega, Southampton, UK).

For gene expression analyses, RNA was extracted using the hot phenol method (Verwoerd et al., 1989), with some modification. The powdered plant material (0.1 g) was suspended in equal volumes of extraction buffer (0.1 M Tris/HCl, 0.1 M LiCl, 1% SDS, 10 mM EDTA pH 8.0) and phenol (total volume 0.4 mL) at 80°C and mixed vigorously for 30 s. Chloroform: isoamyl alcohol mixture (24: 1, total volume 0.5 mL) was added to the sample which was mixed vigorously again for 30 s. Centrifugation at 7200 *g* for 5 min at 4°C separated the phenol from the aqueous phase, which was removed and mixed with an equal volume of chloroform: isoamyl alcohol mixture (24:1). The sample was centrifuged again at 7200 *g* for 5 min at 4°C and the aqueous phase transferred to a clean tube, mixed with an equal volume of 4 M LiCl and left overnight at 4°C to allow precipitation of the RNA, which was subsequently collected by centrifugation at 7200 *g* for 20 min at 4°C. The pellet was washed with 80% ethanol to remove any residual salts and any contaminating DNA was removed using DNaseI (Promega, Southampton, UK), following the manufacturer’s protocol. Further purification was achieved by extraction with an equal volume of phenol: chloroform: isoamyl alcohol (25:24:1). The aqueous phase was retained, mixed with one tenth volume of 3 M NaOAc and 2.5 volumes of ethanol, and placed at -80°C to allow the RNA to precipitate. The RNA was then collected by centrifugation for 20 min at 7200 *g* at 4°C. The supernatent was removed and the pellet was dried and re-dissolved in diethylpyrocarbonate (DEPC)-treated water.

### Amplification and Cloning of Polymerase Chain Reaction (PCR) Products

cDNA was generated from the total RNA using either SuperScript II or Superscript III reverse transcriptase (Invitrogen, Paisley, UK). The primers used to amplify PCR products corresponding to genes involved in asparagine metabolism are given in **Supplementary Table S1**. Products were amplified using Phusion DNA Polymerase (Finnzymes, Vantaa, Finland), which has proof-reading activity, using 1 μL of cDNA with primers at a final concentration of 0.5 μM each (except in the case of degenerate primers where the final concentration was 1 μM). Dimethylsulfoxide was added to a final concentration of 3% to assist denaturation of the template.

Primer optimisation was performed for all primer pairs using a gradient of annealing temperatures, but typical cycling conditions were: initial denaturation at 98°C for 30 s, followed by 35 cycles at 98°C for 10s, 60°C for 30 s, 72°C for 30 s, and a final extension at 72°C for 10 min. PCR products were purified using the Wizard PCR Clean-up system (Promega, Southampton, UK) and A-tailed using Taq polymerase. They were then ligated into the pGEM-T Easy Vector (Promega, Southampton, UK) and the resulting recombinant plasmids transformed into XL-10 Gold competent cells (Agilent Technologies, Wokingham, UK). After selection and liquid culture of recombinant colonies, plasmid DNA was purified using the QiaQuick MiniPrep kit (Qiagen Ltd, Crawley, UK).

Rapid amplification of cDNA ends (RACE) was used to extend the *ASN1* sequence in the 5′ and 3′ direction, to complete the 5′ end of the *GCN2* sequence, and for the aspartate kinase sequence to extend it in the 5′ direction into the leader sequence and complete it at the 3′ end. The primers used for RACE are given in **Supplementary Table S2**. Total RNA was diluted to a concentration of 0.5 μg/μL and RACE was performed using a Generacer kit (Invitrogen, Paisley, UK). The PCR reactions used Platinum Pfx DNA polymerase (Invitrogen, Paisley, UK), which has a proof-reading activity to ensure accurate amplification. Touchdown PCR was used to increase the specificity of the amplification, with an initial step at 94°C for 2 min to denature the template and to release the polymerase from a thermolabile inhibitor. This was followed by five cycles of 30 s at 94°C for denaturation, followed by 72°C for 1 min/kb for annealing and extension. The annealing/extension temperature was decreased to 70°C for the next five cycles. There were then 25 cycles of denaturation for 30 s at 94°C, annealing at 65–68°C for 1 min/kb (temperature determined by annealing temperature of gene-specific primers) and extension at 68°C for 1 min/kb. The final extension step was at 68°C for 10 min.

RACE products were separated by electrophoresis, purified using S.N.A.P^TM^ columns (Invitrogen, Paisley, UK) and ligated into the Zero Blunt TOPO vector (Invitrogen, Paisley, UK). Recombinant plasmids were used to transform OneShot TOP10 competent cells (Invitrogen, Paisley, UK). Colonies were transferred to liquid culture and grown for 24 h at 37°C before plasmid DNA was prepared using a QiaQuick MiniPrep kit (Qiagen Ltd, Crawley, UK).

### Nucleotide Sequence Analysis

Nucleotide sequence analysis was performed by MWG Biotech (Wolverhampton, UK) and contigs were assembled using ContigExpress or Geneious^1^ (Kearse et al., 2012). The basic alignment search tool (BLAST) was used to identify homologous proteins in the non-redundant protein database *via* the National Center for Biotechnology Information portal^2^. Amino acid sequence alignments were performed using Geneious version 8^1^ (Kearse et al., 2012) or the alignment tools provided by EMBL-EBI^3^.

### Expression Analysis

Leaf samples were taken pre-anthesis, then both leaf and grain samples were taken at 7, 14, 21, and 28 dpa. The sampled material was flash-frozen in liquid nitrogen and stored at -80°C before analysis. The gene-specific primers used in the quantitative PCR (qPCR) analysis are given in **Supplementary Table S3**. The reference genes used for the analysis were based on those previously used in a qPCR experiment in wheat (Byrne et al., 2012): glyceraldehyde 3-phosphate dehydrogenase (*GAPDH*) (rye expressed sequence tag (EST) database, accession number BG263908); succinate dehydrogenase subunit 3 (*SDH*) (wheat gene, GenBank, accession number AF475119); cell division control protein (*CDC*) (rye EST database, accession numbers BE704894 and BE587777) (primers designed by Giménez et al. (2011) for analysis of gene expression in barley).

Primer efficiency was tested with a cDNA dilution series, with a no-template control included to ensure there was no DNA contamination or primer dimer formation. Technical replicates were performed in triplicate to ensure repeatability. Percentage efficiency (E) of a primer pair was calculated using the equation:

$$E=(10^{\frac{-1}{m}}-1)*100$$

where *m* is the slope of the line produced by plotting the mean threshold cycle (*ct*) values against the log transformed cDNA concentration. A line with slope -3.322 gives an efficiency of 100%. Primer pairs with efficiencies between 90 and 110% were deemed acceptable for use in gene expression analysis experiments. Melt curves were produced to ensure single products were amplified and standard deviations of *ct* values for technical replicates were analyzed to ensure repeatability of amplification curves. Melt curves were produced by heating final qPCR products at a temperature gradient between 60 and 95°C, a single fluorescence peak indicating a single amplicon.

First-strand cDNA synthesis was performed using SuperScript III (Invitrogen) and 0.5–1.0 μg of total RNA. The RNA was primed with an anchored dT20 primer (Invitrogen) in a final volume of 20 μL. The qPCR reaction mixture consisted of 10 μL SYBR Green JumpStart Taq ReadyMix (Sigma, Poole, UK), 1 μL diluted cDNA and primers (final concentration of 250 or 500 nM, whichever was shown to be optimal). Samples were run in an ABI 7500 real-time PCR system (Applied Biosystems, Foster City, CA, USA) and the amplification conditions were 95°C for 10 min, then 40 cycles at 95°C for 15 s followed by 60°C for 1 min. The efficiencies of the reactions were estimated using the LinReg PCR program (Ramakers et al., 2003). This uses fluorescence data and *ct* values to fit a linear regression to the reaction curve for each reaction well. Ideally, the reaction efficiency should be 2, representing a doubling of transcript copies with each PCR cycle. Individual reaction efficiencies were compared to confirm that there was no effect of experimental treatment on the reaction efficiency. Mean efficiency values per gene were therefore suitable for use in further calculations. The *ct* (at threshold fluorescence) and efficiency values were used to calculate the normalised relative quantity (NRQ) of transcripts with respect to the reference genes: *CDC*, *SDH*, and *GAPDH*, for each sample/target gene combination. The NRQ was calculated according to the following equation:

$$NRQ=\frac{E_{t\,\;\arg\,\;et}^{-ct,t\,\;\arg\,\;et}}{\sqrt{E_{SDH}^{-ct,SDH}}*E_{GAPDH}^{-ct,GAPDH}*E_{CDC}^{ct,CDC}}$$

where *E_target_*, *E_SDH_, E_GAPDH_*, and *E_CDC_* are the mean estimated reaction efficiencies for a particular target gene and the three reference genes, and where ‘*ct,target’*, ‘*ct,SDH’, ‘ct,CDC’*, and ‘*ct,GAPDH’* are the corresponding *ct* values. Statistical analysis of the gene expression data was performed using the GenStat^®^ (2010, Thirteenth Edition; VSN International Ltd, Hemel Hempstead, UK) statistical system.

Reference gene stability over the treatments (S, N, or time) was confirmed using *t*-tests on the *ct* values to verify suitability for their inclusion in the calculation of NRQ. ANOVA was then applied to the log_2_ transformed inverse of the NRQ data. This transformation was performed to ensure homogeneity of variance across treatments, and to provide values on the *ct*-scale. With log_2_(1/NRQ) values, as with *ct* values, a higher value indicates low gene expression and a lower value represents higher gene expression (Rieu and Powers, 2009). The ANOVA was performed to test (*F*-tests) statistical significance of treatment effects (S, N, or time) on gene expression, accounting for variation among block replicates which is not possible to do using a *t*-test.

## Results

### Free Amino Acid Concentrations in the Grain of Rye Plants Grown with Different Combinations of Nitrogen and Sulfur Availability

A previous study with field-grown rye showed a significant effect of nitrogen availability on free amino acid, and particularly free asparagine, concentration in rye grain, with increased nitrogen availability bringing about a rise in free asparagine concentration (Postles et al., 2013). Sulfur deficiency has been shown to cause a massive accumulation of free asparagine and to a lesser extent free glutamine in wheat grain (Muttucumaru et al., 2006; Granvogl et al., 2007; Curtis et al., 2009, 2014). However, sulfur fertilization had only a marginal effect in field-grown rye, mitigating the effect of increased nitrogen in some varieties (Postles et al., 2013). In the present study, rye plants of three varieties, Agronom, Festus, and Askari, were grown in pots containing vermiculite so that they were dependent on supplied nutrients and a more severe degree of sulfur deficiency could be imposed, in combination with more precisely controlled levels of nitrogen. The interaction of nitrogen and sulfur supply was investigated by supplying four treatments in a randomized block design: full nitrogen and sulfur (N^+^S^+^); full nitrogen, no sulfur (N^+^S^-^); 5% nitrogen, full sulfur (N^-^S^+^); and 5% nitrogen, no sulfur (N^-^S^-^).

The Agronom plants did not produce enough grain for inclusion in the analysis, but sufficient material was sampled from Festus and Askari at 21 and 28 dpa. The grain was ground to a fine powder and free amino acids were analyzed by GC-MS, following derivatisation. The full dataset is given in **Supplementary Table S4**. Analysis of variance (ANOVA) was applied to the data and showed that both nitrogen and sulfur availability had an effect on the accumulation of free asparagine.

**FIGURE 1:**
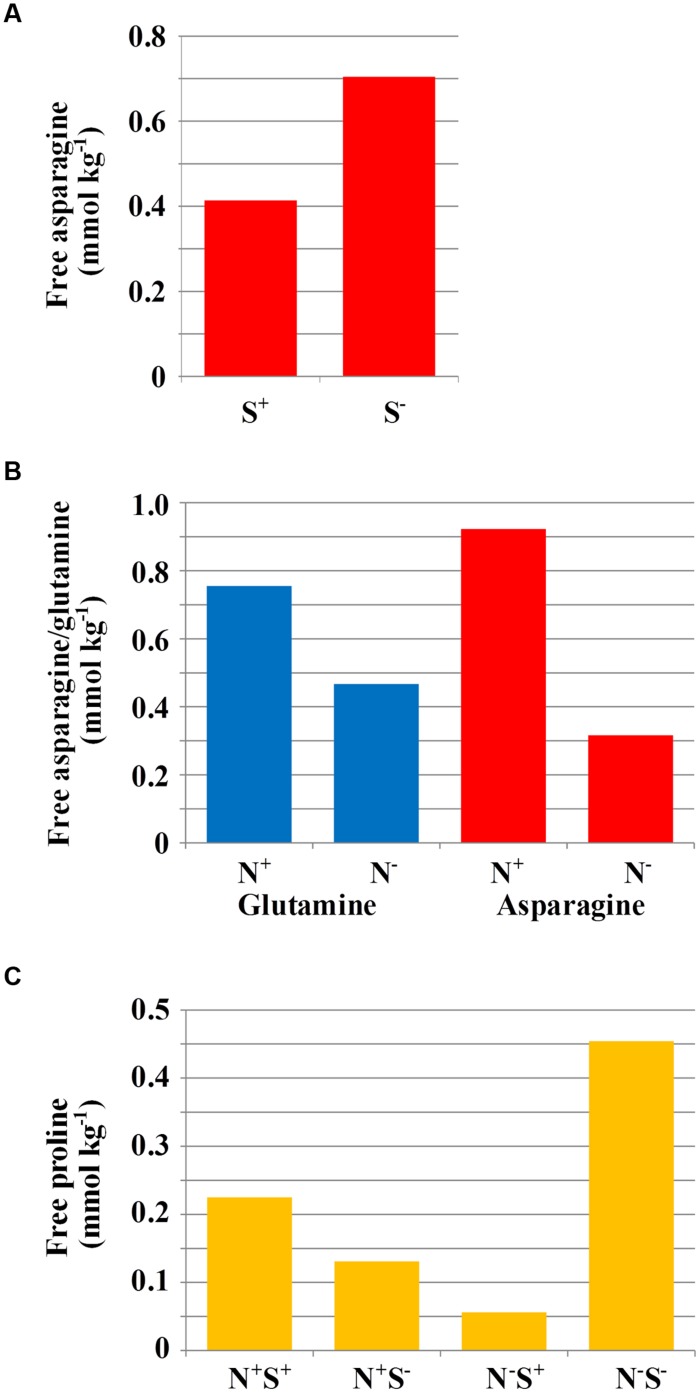
**(A)** Mean free asparagine concentration (mmol kg^-1^) in grain of rye grown under different sulfur treatments (*n* = 24). For statistical analysis see **Table 1A**. **(B)** Mean free glutamine and asparagine concentration (mmol kg^-1^) in grain of rye grown in a glasshouse under different nitrogen treatments (*n* = 24). For statistical analysis see **Table 1B**. **(C)** Mean free proline concentration (mmol kg^-1^) in grain of rye varieties grown in a glasshouse under different combinations of nitrogen and sulfur treatments (*n* = 12). For statistical analysis see **Table 1C**.

Under sulfur deficiency, plants of both varieties accumulated more free asparagine than plants grown with sufficient sulfur (*F*-test, *p* = 0.004). The means of log_e_ transformed data from the ANOVA are given in **Table 1A**, and the means of the raw data are shown graphically in **Figure 1A**. The concentration of both free asparagine and free glutamine increased significantly in response to nitrogen application (*F*-test, *p* < 0.001 for free asparagine; *p* = 0.004 for free glutamine). The means of the raw data are shown graphically in **Figure 1B** and the means of the log_e_ transformed data are given in **Table 1B**. The ANOVA took into account the design of the experiment and tested main effects of nitrogen and sulfur separately whilst also testing for interaction effects, which were not significant (*F*-tests, *p* > 0.05).

**Table 1:** Log*_e_* transformed free amino acid concentrations in rye grain grown under nutrient treatments [sulfur sufficiency (S^+^) and deficiency (S^-^); nitrogen sufficiency (N^+^), and deficiency (N^-^)], in different varieties and developmental stages, as indicated.

(A) Free asparagine: effect of sulfur treatment (*n* = 24).
**Treatment**	**Log_e_ (Asn)**

S^+^	-0.88
S-	-0.35

*SED = 0.148 on 12 df; LSD (5%) = 0.322.*			

**(B) Free asparagine and glutamine: effect of nitrogen treatment (*n* = 24).**

**Treatment**	**Log*_e_* (Asn)**	**Log*_e_* (Gln)**

N^+^	-0.08	-0.28
N^-^	-1.15	-0.76

*Asn: SED = 0.148 on 12 df; LSD (5%) = 0.322.*			
*Gln: SED = 0.136 on 12 df; LSD (5%) = 0.296.*			

**(C) Free proline: Effect of sulfur interacting with nitrogen (*n* = 12).**

	**Sulfur**	**Log*_e_* (Pro)**
		
**Nitrogen→**		**N^+^**	**N^-^**

	S^+^	-1.49	-2.88
	S^-^	-2.03	-0.79

*SED = 0.689 on 12 df; LSD (5%) = 1.501.*			

**(D) Free asparagine, proline and and total free amino acids: Effect of variety (*n* = 24).**

**Variety**	**Log*_e_* (Asn)**	**Log*_e_* (Pro)**	**Log*_e_* (Total)**

Askari	-0.86	-2.75	2.53
Festus	-0.37	-0.84	2.66

*Asn: SED = 0.148 on 12 df; LSD (5%) = 0.322.*			
*Pro: SED = 0.487 on 12 df; LSD (5%) = 1.062.*			
*β-ABA: SED = 0.969 on 12 df; LSD (5%) = 2.111.*			
*Total: SED = 0.052 on 12 df; LSD (5%) = 0.114.*			

**(E) Free glutamine: Effect of variety interacting with sulfur and developmental stage (*n* = 6).**

**Variety**	**Treatment**	**Log*_e_* (Gln)**
		
		**21 dpa**	**28 dpa**

Askari	S^+^	-0.45	-0.85
	S^-^	-0.69	-0.96
Festus	S^+^	-0.39	-0.02
	S^-^	0.12	-0.88

*Standard error of the difference (SED) for comparisons:*			
*1. Means with same variety and treatment: SED = 0.238 on 11 df; LSD (5%) = 0.524.*			
*2. All other comparisons: SED = 0.256 on 22 df; LSD (5%) = 0.529.*			

**(F) Free glutamate: Effect of variety interacting with nitrogen treatment and developmental stage (*n* = 6).**

**Variety**	**Treatment**	**Log*_e_* (Glu)**
		
		**21 dpa**	**28 dpa**

Askari	N^+^	-1.14	-6.90
	N^-^	-4.35	-6.64
Festus	N^+^	-1.54	-2.18
	N^-^	0.34	-6.08

*Standard error of the difference (SED) for comparisons:*
*1. Means with same variety and nutrient treatment: SED = 1.950 on 11 df; LSD (5%) = 4.292.*			
*2. All other comparisons: SED = 2.050 on 22 df; LSD (5%) = 4.242.*			

**(G) Free glycine and isoleucine: Effect of developmental stage (*n* = 24).**

**Developmental stage**	**Log*_e_* (Gly)**	**Log*_e_* (Ile)**

21 dpa	-1.43	-2.72
28 dpa	-3.31	-4.83

*Gly: SED = 0.677 on 11 df; LSD (5%) = 1.490.*			
*Ile: SED = 0.700 on 11 df; LSD (5%) = 1.542.*			

**(H) Free methionine: Effect of sulfur treatment interacting with developmental stage (*n* = 6).**

**Variety**	**S**	**Log*_e_* (Met)**
		
		**21 dpa**	**28 dpa**

Askari	S^+^	-3.87	-4.38
	S^-^	-4.38	-4.34
Festus	S^+^	-3.98	-3.99
	S^-^	-3.89	-4.61


*Standard error of the difference (SED) for comparisons:*

*1. Means with same variety and nutrient treatment: SED = 0.190 on 11 df; LSD (5%) = 0.418.*

*2. All other comparisons: SED = 0.312 on 17 df; LSD (5%) = 0.658.*

There were also effects of nutrient supply on other free amino acids: The concentration of free proline, for example, showed significant (*F*-test, *p* = 0.019) effects of nitrogen interacting with sulfur. When sulfur was available, there was a significant decrease (LSD, *p* < 0.05) in grain proline concentration when nitrogen was low, compared to when nitrogen was sufficient. However, reduced sulfur application led to an accumulation of proline when nitrogen was low. The raw data means are shown graphically in **Figure 1C** and the log_e_ transformed data means from ANOVA are given in **Table 1C**.

The analysis also showed an effect of variety, with Festus accumulating significantly more (*F*-test, *p* = 0.006) free asparagine than Askari (**Table 1D**), although there was no interaction effect between variety and nutrient treatments, so both varieties responded to nutrient availability in the same manner. Festus also accumulated more free proline than Askari (*F*-test, *p* = 0.002). Conversely, the total free amino acid concentration was significantly higher in Festus than Askari (*F*-test, *p* = 0.029).

Glutamine accumulation was also affected by variety but in interaction with sulfur application and developmental stage (*F*-test, *p* = 0.010, **Table 1E**). At 28 dpa, there was significantly more (LSD, *p* < 0.05) glutamine in Festus than Askari when sulfur was sufficient, and at 21 dpa when sulfur was deficient. In Festus, when sulfur was limiting there was more glutamine in 21 dpa grain than in 28 dpa grain (**Table 1E**).

There was a significant interaction effect between variety, nitrogen and developmental stage on the accumulation of glutamate (*F*-test, *p* = 0.037, **Table 1F**). More glutamate accumulated in Askari grain at 21 dpa than 28 dpa when nitrogen was sufficient. There was more glutamate at 21 dpa in Festus than Askari under nitrogen deficiency, and there was more glutamate in Festus grain at 21 dpa than 28 dpa when nitrogen was low.

There was an effect of developmental stage on the accumulation of both free glycine and free isoleucine (**Table 1G**), with significantly more in grain sampled at 21 dpa than 28 dpa (*F*-test, *p* < 0.05 for both). There was also an interaction effect between sulfur treatment, variety and developmental stage (*F*-test, *p* = 0.007) on the accumulation of free methionine (**Table 1H**), with grain of Askari plants accumulating more free methionine at 21 dpa than 28 dpa when sulfur was sufficient, but grain of Festus plants accumulating more methionine at 21 dpa when sulfur was deficient (LSD, *p* < 0.05).

### Molecular Cloning of cDNAs Corresponding to Genes Involved in Asparagine Metabolism

While the free amino acid data revealed a number of interesting responses to the nutrient treatments it was the genetic basis of the changes in free asparagine concentration that were the main focus of the present study, with genes affecting glutamine and aspartate also of interest because free asparagine is synthesized *via* the amidation of aspartate using an ammonium group from glutamine. Specifically, it was decided to analyze the expression of genes encoding asparagine synthetase-1, which has been shown to respond to sulfur availability in wheat (Gao et al., 2016), asparaginase, aspartate kinase, and cytosolic GS1. In addition, genes encoding protein kinases SnRK1 and GCN2, both of which have been implicated in the regulation of asparagine synthetase gene expression (Baena-Gonzalez et al., 2007; Byrne et al., 2012) were included.

The nucleotide sequences of asparagine synthetase-1 genes from wheat (*TaASN1*, GenBank AY621539), barley (*HvAS1*, GenBank AF307145), and maize (GenBank BT035878) were used to design primers (**Supplementary Table S1**) for amplification of an asparagine synthetase PCR product from RNA of rye seedlings, cv. Askari. Rapid amplification of cDNA ends (RACE) was used in both the 5′ and 3′ directions to generate nucleotide sequence data for the complete coding region. A proof-reading polymerase was used for the amplification and a single PCR product was cloned and given the name *ScASN1*. It encodes a 585 amino acid, 65.43 kDa protein with more than 99% amino acid sequence identity with wheat and barley asparagine synthetase proteins derived from GenBank accession numbers AAU89392.1 and AAK49456.1, respectively. The nucleotide sequence has been submitted to GenBank and given the accession number KU853956.

The nucleotide sequence of a barley gene (GenBank AF308474) was used to design primers to amplify a K^+^-dependent asparaginase-encoding cDNA. Three distinct PCR products were obtained and given the names *ScASP1*, *ScASP2*, and *ScASP3*; they have been assigned accession numbers KU853957, KU853958, and KU853959, respectively. They encode proteins of 333 to 340 amino acids, 35.04 to 35.76 kDa, showing 95.6 to 98.8% amino acid sequence identity with each other and 94.3 to 94.9% with the barley protein. *ScASP1* and *ScASP2* both contain a 21-nucleotide duplication at the 5′ end, resulting in the encoded proteins having a seven amino acid repeat at the N-terminal end, assuming that the first ATG is used as the translation start site. *ScASP1* and *ScASP2* were amplified and cloned independently, and have several polymorphisms elsewhere in the sequence (they show 93.5% identity at the nucleotide level). We therefore conclude that the repeat is not an artifact and that it is present in more than one asparaginase gene in rye.

No monofunctional aspartate kinase (ASK) sequence had been published for either wheat or barley, but a BLAST search of wheat genome data^4^ using nucleotide sequences from rice (*Oryza sativa*) (GenBank NM_001051806.1) and maize (GenBank EF424250) identified several separate contigs that could be assembled into a complete ASK-encoding sequence. This contig showed high similarity to a wheat sequence in an unannotated GenBank entry, AK333665, and to a UniGene database entry, Hv6529, comprising several barley expressed sequence tags. Primers were therefore designed from an alignment of nucleotide sequences from AK333665 and Hv6529 and used to amplify and clone a rye ASK PCR product, *ScASK1*. This was extended to the 3′ end using RACE, and the entire nucleotide sequence submitted to GenBank (Accession KU853960). *ScASK1* encodes a protein of 52.64 kDa showing 99.4% amino acid sequence identity with that of A333665, and 92.8 and 93.6%, respectively, with the ASK1 proteins of maize (EF424250) and rice (NM_001051806.1).

Cytosolic glutamine synthetase is typically encoded by a small family of genes. In wheat, for example, seven cytosolic *GS1* genes have been identified: *GS1a*, *GS1b*, and *GS1c* (GenBank AAZ30057.1, AAZ30058.1, and AAZ30059.1) as well as more divergent forms, *GSr1* and *GSr2* (GenBank AY491968 and AY491969) and *GSe1* and *GSe2* (GenBank AY491970 and AY491971). At the time, only one *GS1* transcript had been identified in barley, *GS1-1* (GenBank X69087.1), although additional sequences have since been added. An alignment of the nucleotide sequence of this transcript with those of wheat *GS1a*, *b*, and *c*, was used to design primers to amplify a homologous sequence from rye. Three almost identical *GS1*-like PCR products were amplified and their nucleotide sequences determined. They were found to encode identical proteins of 356 amino acid residues, with a molecular weight of 39.11 kDa, showing 97.2% amino acid sequence identity with wheat GS1a and GS1c, 97.5% with wheat GS1b and 96.6% with barley GS1. One of the sequences, *ScGS1*, has been submitted to GenBank and given the accession number KU853962. By this time, another rye GS1 sequence had been added to the GenBank database, accession number AFB69879.1. It encodes a protein showing only one residue mismatch with the ScGS1 protein.

Lastly, five overlapping PCR products were amplified using primers designed from the wheat GCN2-type gene, *TaGCN2* (GenBank FR839672). The nucleotide sequences of the five products were assembled into a single contig of 4269 bp, which was submitted to GenBank and assigned accession number KU853961. The ATG translation start codon was not present and attempts to amplify more of the 5′ region of the transcript by RACE were unsuccessful. Assuming that the 5′ end of the coding region is similar to that of *TaGCN2*, the derived amino acid sequence was missing a methionine and a glycine residue at the N-terminal end. Otherwise, the derived amino acid sequences of ScGCN2 and TaGCN2 showed 98.5% identity, and the molecular weights were also very similar at 140.54 kDa and 140.12 kDa, respectively. Like TaGCN2, ScGCN2 contains juxtaposed eIF2α kinase and His-tRNA-like regulatory domains that typify GCN2-like protein kinases (Byrne et al., 2012).

### Gene Expression

The expression of the candidate genes described above, along with a previously reported rye gene encoding a SnRK1-type protein kinase (Alderson et al., 1991; GenBank M74113) was studied using quantitative PCR in order to relate gene expression to the accumulation of free amino acids in the grain.

**FIGURE 2:**
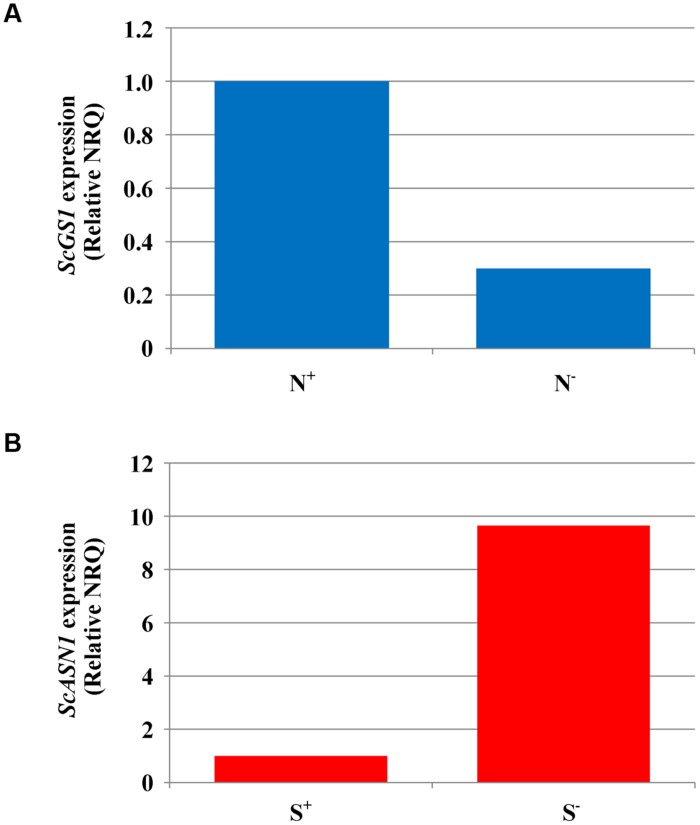
**Gene expression analyses in rye (cv. Askari) grain at 21 dpa.** **(A)** Expression of glutamine synthetase-1 gene (*ScGS1*) in grain produced under different nitrogen treatments (shown as N^+^ and N^-^). Mean normalised relative quantities (NRQ) are shown with the NRQ for the control treatment (N^+^) set at 1 (*n* = 3; log transformed means at N^+^ = -56.18, at N^-^ = -54.70; SED = 0.253 on 2 df, LSD (5%) = 1.09; *F*-test, *p* < 0.05). **(B)** Expression of asparagine synthetase-1 gene (*ScASN1*) in grain produced under different sulfur treatments (shown as S^+^ and S^-^). Mean normalised relative quantities (NRQ) are shown with the NRQ for the control treatment (S^+^) set at 1 (*n* = 3; log transformed mean at S^+^ = -51.59, at S^-^ = -54.00, SED = 0.772 on 2 df; LSD (5%) = 3.323; *F*-test, *p* = 0.089).

Gene expression was studied in grain and flag leaf of variety Askari grown under sulfur deficiency, nitrogen deficiency, and the control treatment of full nutrient sufficiency, collected at 21 and 28 dpa, when enough material could be sampled for analysis. The results from the qPCR experiments detailing the raw NRQ values and the statistical analysis of these values having been transformed back to the *ct*-scale are given in full in **Supplementary Tables S5**–**S9**. In most cases the treatments did not give rise to significant changes in expression of the genes studied, but there was a significant (*F*-test, *p* < 0.05) effect of nitrogen availability on the expression of *ScGS1* in grain sampled at 21 dpa, with expression under nitrogen deficient conditions less than a third of that under nitrogen sufficiency (**Figure 2A**). Similar ratios were obtained at 28 dpa but the result was no longer statistically significant.

There was also some evidence of a real effect of sulfur for *ScASN1*. Expression at 21 dpa was almost 10 times higher under sulfur deficiency than when sulfur was available (**Figure 2B**). However, this result was only marginally significant (*F*-test, *p* = 0.089, **Supplementary Table S5**) and once again the statistical significance was lost at 28 dpa (**Supplementary Table S6**), even though the ratio in expression levels remained at approximately 10:1. The lack of stronger statistical results for these data, despite the apparently large differences in the means, was due to high underlying variation.

## Discussion

We have shown significant effects of nitrogen and sulfur supply on free asparagine and glutamine accumulation in developing rye grain, with nitrogen supply causing an increase in accumulation of both amino acids (by ∼threefold in the case of free asparagine), while sulfur deficiency led to an approximately 70% increase in free asparagine accumulation. Sulfur deficiency causes a massive accumulation of free asparagine in wheat grain (Muttucumaru et al., 2006; Granvogl et al., 2007; Curtis et al., 2009) but this is the first evidence that rye could show a similar, although less dramatic response. Even so, the pot-grown rye in this study was not supplied with any sulfur at all, and wheat treated in the same way can show an up to 30-fold increase in free asparagine concentration, while a field-based study on rye did not show any significant effect of sulfur supply (Postles et al., 2013). Therefore, the response in rye is much less dramatic than that in wheat. The response seen in wheat may be attributed, at least in part, to the use of free asparagine as an alternative grain nitrogen store when wheat is unable to synthesize storage proteins (Curtis et al., 2009). Rye may be better able to scavenge available sulfur from the soil than wheat (Bushuk, 2001), but may also respond differently: Postles et al. (2013) showed that grain nitrogen content was reduced at low levels of sulfur application, even when plenty of nitrogen was available. The results of the two studies suggest that rye responds to sulfur deficiency by accumulating less nitrogen in the grain, only storing nitrogen as free asparagine in response to extreme sulfur deficiency, as was imposed here.

The wheat genome contains four asparagine synthetase genes, showing differential tissue-specific and developmental regulation of gene expression (Gao et al., 2016). Asparagine synthetase-1 (*ASN1*) gene expression has been shown to respond to sulfur deficiency (Byrne et al., 2012; Gao et al., 2016), and the cloning and expression analysis of a rye *ASN1* gene (*ScASN1*) did show an apparent increase in gene expression in response to sulfur deficiency. However, this result was only marginally statistically significant (*F*-test, *p* = 0.089) due to the extent of underlying variation. With hindsight, this variation could have been restrained by having more than three biological replicates. Nevertheless, qPCR is an inherently variable technique, which is the main reason why the data arising need to be transformed to the log_2_(1/NRQ) scale for analysis. While any conclusions must be drawn tentatively given marginal statistical significance, given the fact that wheat *TaASN1* has been shown to respond to sulfur deficiency, we consider it likely that the apparent increase in *ScASN1* expression was real and at least partly responsible for the increase in free asparagine concentration.

While the *GS*-1 gene, *ScGS1-1*, did not respond to sulfur supply, it did show a significant (*F*-test, *p* < 0.05) response to nitrogen availability, with expression when nitrogen was fully supplied more than three times higher than when nitrogen supply was reduced by 95%. It is likely that this was responsible either wholly or in part for the increased accumulation of free glutamine when sufficient nitrogen was supplied, and possibly also indirectly for the increase in free asparagine, the synthesis of which requires an ammonium group from glutamine.

There were no other statistically significant (*F*-test, *p* < 0.05) changes in gene expression in response to the different treatments. However, that does not necessarily mean that the enzymes encoded by the genes that were studied do not play an important role in the regulation of asparagine metabolism. The protein kinases, GCN2 and SnRK1, in particular, both of which have been implicated in the regulation of asparagine synthetase gene expression (Baena-Gonzalez et al., 2007; Byrne et al., 2012), may be regulated by post-transcriptional mechanisms.

The treatments also had significant effects on other free amino acids, with proline, for example, showing effects of nitrogen availability interacting with sulfur. Indeed, a major outcome of the study was to reinforce the conclusion that nutrient availability can have a profound impact on the concentrations of different free amino acids, a concept discussed in more depth by Halford et al. (2015). Free amino acids are sometimes overlooked by plant physiologists when considering grain composition because most of the nitrogen in the grain is incorporated into proteins. This oversight is unfortunate and must be rectified given the importance of free amino acids for flavor, color, and aroma development during cooking and processing, as well as in the production of undesirable contaminants such as acrylamide.

## Author Contributions

JP, conducted approximately 90% of the experimentation and 20% of paper writing. TC, conducted approximately 10% of the experimentation and 5% of paper writing. SP, responsible for statistical analyses, 10% of project planning and 5% of paper writing. JE, jointly responsible with DM for supervision of analytical chemistry, plus 5% of project planning and 5% of paper writing. DM, jointly responsible with JE for supervision of analytical chemistry, plus 10% of project planning and 5% of paper writing. NH, project leader, responsible for 75% of project planning and 60% of paper writing. All authors have approved the final version of the manuscript, and are accountable for its content.

## Disclaimer

The project was supported by a number of companies and organizations from the rye and wheat supply chains.

## Conflict of Interest Statement

The authors declare that the research was conducted in the absence of any commercial or financial relationships that could be construed as a potential conflict of interest.

## Footnotes

1 http://www.geneious.com

2 http://www.ncbi.nlm.nih.gov/

3 http://www.ebi.ac.uk/Tools/psa/

4 http://www.cerealsdb.uk.net/
