# Supplementary material for: Changes in Free Amino Acid Concentration in Rye Grain in Response to Nitrogen and Sulfur Availability, and Expression Analysis of Genes Involved in Asparagine Metabolism
Source: Front Plant Sci. 2016 Jun 22;7:917. doi: 10.3389/fpls.2016.00917 (PMC4916186; doi:10.3389/fpls.2016.00917)
Supplement: Supplementary file 1 [file Table_1.DOCX]

**Changes in free amino acid concentration in rye grain in response to nitrogen and sulfur availability, and expression analysis of genes involved in asparagine metabolism**

***Jennifer Postles^1**^, Tanya Y. Curtis^1^, Stephen J. Powers^2^, J. Stephen Elmore^3^, Donald S. Mottram^3^, and Nigel G. Halford^1*^***

*^1^Plant Biology and Crop Science Department, Rothamsted Research, Harpenden, Hertfordshire AL5 2JQ, United Kingdom*

*^2^Computational and Systems Biology Department, Rothamsted Research, Harpenden, Hertfordshire AL5 2JQ, United Kingdom*

*^3^Department of Food and Nutritional Sciences, University of Reading, Whiteknights, Reading, RG6 6AP, UK*

**Supplementary Data**

**Table S1.** Nucleotide sequences of primers designed for amplification of polymerase chain reaction (PCR) products corresponding to rye genes involved in asparagine metabolism. F and R in the primer name indicate ‘forward’ and ‘reverse’ primers. All sequences are shown in the 5’ to 3’ direction. Standard degeneracy codes are used.

| **Gene** | **Primer** | **Nucleotide sequence** |
| --- | --- | --- |
| Asparagine synthetase 1 (*ASN1*) | ScAS1F | ATGTGYGGCATMCTSGCDGTSC |
|  | ScAS2JR | GGAGWTKYYCCTGYTCATASGC |
| Asparaginase (*ASP*) | ASP F | ATGGCGCGCTGGGCCATTGC |
|  | ASP R | TCACTCCCAGATGCCGACCT |
| Aspartate kinase (*AK*) | AK F2 | ATGAAGTTCGGCGGCTCGT |
|  | AK R2 | TTCACCTTGGACGCCCCTT |
| Glutamine synthetase 1 (*GS1*) | GS1 F | ATGGCGCTCCTCACCGATCTC |
|  | GS1 R | GGGCTTCCACAGGATGGTGG |
| General control nonderepressible-2 (*GCN2*) | GCN2 F1 | GCACAGCGCGAGGAAGAAGA |
|  | GCN2 R1 | AGCCCCCCTGACCAAGTGAA |
|  | GCN2 F2 | ATGCGCCATCTCATGTTTC |
|  | GCN2 R2 | TCAATCGCAGATGGACGTT |
|  | GCN2 F3 | TAGAACAGAAGTGGCCACAGAT |
|  | GCN2 R3 | AAGAGCCTTGCAAGCAGATT |
|  | GCN2 F4 | CTGAATCACAGCAAACTCGC |
|  | GCN2 R4 | GCTTCAGACAGGAACTTCACAA |
|  | GCN2 F5 | ATGATAAATCTGCTTGCAAGGC |
|  | GCN2 R5 | GCATTCCCTATGAGCATCAAA |

**Table S2.** Nucleotide sequences of gene-specific primers designed for the amplification of genes of interest from rapid amplification of cDNA ends (RACE)-ready cDNA. All sequences are shown in the 5’ to 3’ direction.

| **Gene** | **Primer** | **Nucleotide sequence** |
| --- | --- | --- |
| Asparagine synthetase 1 (*ASN1*) | ASN1 GSP R1 | GTCCCCCAGCGCTTTGCAGCCTTT G |
|  | ASN1 GSP F1 | GCATGTAGCACGGCGAAGGCGGTAGA |
| General control nonderepressible-2 (*GCN2*) | GCN2 GSP R1 | CGCAACTTTGGGCACTTGTGGGGGTAA |
|  | GCN2 Nested R | GAAAAATTGCAGGCGCCGTTAG |
| Aspartate kinase (*AK*) | AK GSP F1 | GCAGTCGTTCATCTCCTACAGCACAGAT |
|  | AK GSP R1 | ACGACACCGACGAGCCGCCGAACTT |
|  | AK GSP R2 | GGTCAAGTCACTGCCGCCCCTTCCTAA |

**Table S3.** Nucleotide sequences of gene-specific primers used for quantitative polymerase chain reaction (qPCR) experiments. All sequences are shown in the 5’ to 3’ direction.

| **Gene** | **Primer** | **Nucleotide sequence** |
| --- | --- | --- |
| Asparagine synthetase 1 (*ASN1*) | ASN1 F5 | GCTTTCGAAAAGGCTGTCAC |
|  | ASN1 R3 | GAGTCAAGGCCACCAGAGAG |
| Aspartate kinase (*AK*) | ASK F1 | GCACAGGTTTTGCATCCCCAATC |
|  | ASK R1 | GCCAGGTGCATGACGGTTATATGA |
| General control nonderepressible-2 (*GCN2*) | GCN2 F1 | CGGGACTTGACACCTAGCAACAT |
|  | GCN2 R1 | CGCACTGCTGAGAGCTCTATGTA |
| Asparaginase (*Asp*) | ASP F4.1 | CATGGACAAGTCTCCCCACT |
|  | ASP R4 | CCGACGTTCTCCTCTGTGAT |
| Glutamine synthetase-1 (*GS1*) | GS1 F4 | GTGCTGGTGCTCACACAAAC |
|  | GS1 R4 | GATGTGCTCCTTGTGCTTCA |
| SNF1-related protein kinase-1 (*SnRK1*) | SnRK1 F2 | GGACCTCAGCTCCTCTTCCT |
|  | SnRK1 R2 | CACTGCACGGGACAGTCTTA |
| Glyceraldehyde 3-phosphate dehydrogenase (*GAPDH*) | GAPDH F1 | ACCCCTTCATCACCACCGACTACATGACC |
|  | GAPDH R1 | GGATCTCCTCAGGGTTCCTGCAGCC |
| Succinate dehydrogenase subunit 3 (*SDH*) | SDH F1 | GAGACGCTCCATTTGCTCTCCGTG |
|  | SDH R1 | ATCTTCTGTCGCAGAGCTCCTAAGGG |
| Cell division control protein (*CDC*) | CDC F1 | CAGCTGCTGACTGAGATGGA |
|  | CDC R1 | ATGTCTGGCCTGTTGGTAGC |

**Table S4.** Free amino acid concentration (mmol/kg fresh weight) in grain grown in the glasshouse under different nutrient treatments. B = experimental block, P = pot, Var = variety, N = nutrient treatment (1 = N^+^S^+^, 2 = N^+^ S^-^, 3 = N^-^ S^+^, 4 = N^-^ S^-^). DPA =days post anthesis.

| **B** | **P** | **Var** | **N** | **DPA** | **Asn** | **Gln** | **Asp** | **Glu** | **Thr** | **Ala** | **Gly** | **Val** | **B-ABA** | **Ile** | **Ser** | **Pro** | **Met** | **Phe** | **Tyr** | **Orn** | **His** | **Trp** | **Total amino acids** |
| --- | --- | --- | --- | --- | --- | --- | --- | --- | --- | --- | --- | --- | --- | --- | --- | --- | --- | --- | --- | --- | --- | --- | --- |
| 1 | 2 | Fes | 3 | 21 | 0.18 | 0.30 | 0.86 | 0.02 | 0.03 | 0.77 | 0.09 | 0.00 | 0.28 | 0.06 | 0.56 | 0.11 | 0.01 | 0.08 | 6.36 | 0.12 | 0.14 | 0.08 | 11.03 |
| 1 | 2 | Fes | 3 | 28 | 0.39 | 0.74 | 0.05 | 0.00 | 0.03 | 1.92 | 0.01 | 0.00 | 0.00 | 0.00 | 0.28 | 0.16 | 0.01 | 0.07 | 6.36 | 0.12 | 0.14 | 0.08 | 12.70 |
| 1 | 3 | Fes | 4 | 21 |  |  |  |  |  |  |  |  |  |  |  |  |  |  |  |  |  |  |  |
| 1 | 3 | Fes | 4 | 28 | 0.59 | 0.25 | 0.36 | 0.34 | 0.03 | 1.20 | 0.07 | 0.00 | 0.00 | 0.00 | 0.00 | 0.20 | 0.01 | 0.07 | 6.34 | 0.12 | 0.14 | 0.08 | 11.24 |
| 1 | 4 | Ask | 4 | 21 | 0.14 | 0.43 | 0.39 | 0.58 | 0.03 | 0.90 | 0.27 | 0.00 | 0.27 | 0.01 | 0.15 | 0.13 | 0.01 | 0.08 | 6.36 | 0.12 | 0.14 | 0.08 | 11.19 |
| 1 | 4 | Ask | 4 | 28 | 0.38 | 0.35 | 0.27 | 0.00 | 0.03 | 1.84 | 0.17 | 0.00 | 0.28 | 0.05 | 0.36 | 0.19 | 0.02 | 0.08 | 6.34 | 0.12 | 0.14 | 0.08 | 12.98 |
| 1 | 5 | Fes | 1 | 21 | 0.71 | 1.02 | 1.29 | 0.13 | 0.03 | 0.94 | 0.22 | 0.05 | 0.00 | 0.19 | 0.17 | 0.47 | 0.02 | 0.11 | 6.35 | 0.12 | 0.14 | 0.08 | 13.26 |
| 1 | 5 | Fes | 1 | 28 |  |  |  |  |  |  |  |  |  |  |  |  |  |  |  |  |  |  |  |
| 1 | 6 | Fes | 2 | 21 | 1.86 | 1.40 | 1.56 | 0.50 | 0.02 | 1.18 | 0.58 | 0.00 | 0.27 | 0.06 | 1.12 | 0.22 | 0.01 | 0.09 | 6.36 | 0.12 | 0.14 | 0.08 | 17.06 |
| 1 | 6 | Fes | 2 | 28 | 1.76 | 0.45 | 0.54 | 0.48 | 0.03 | 0.92 | 0.34 | 0.00 | 0.28 | 0.00 | 0.32 | 0.08 | 0.01 | 0.08 | 6.35 | 0.12 | 0.14 | 0.08 | 13.11 |
| 1 | 7 | Ask | 1 | 21 | 0.76 | 0.74 | 0.95 | 0.11 | 0.00 | 0.65 | 0.19 | 0.00 | 0.28 | 0.07 | 0.08 | 0.22 | 0.02 | 0.09 | 6.35 | 0.12 | 0.14 | 0.08 | 11.69 |
| 1 | 7 | Ask | 1 | 28 | 0.31 | 0.35 | 0.04 | 0.00 | 0.00 | 1.39 | 0.05 | 0.00 | 0.00 | 0.00 | 0.00 | 0.10 | 0.01 | 0.07 | 6.37 | 0.12 | 0.14 | 0.08 | 10.74 |
| 1 | 10 | Ask | 2 | 21 | 0.52 | 0.31 | 0.87 | 0.31 | 0.00 | 0.79 | 0.83 | 0.00 | 0.27 | 0.10 | 0.00 | 0.04 | 0.01 | 0.09 | 6.36 | 0.12 | 0.15 | 0.08 | 11.88 |
| 1 | 10 | Ask | 2 | 28 | 3.97 | 0.83 | 1.46 | 0.03 | 0.00 | 0.98 | 0.75 | 0.07 | 0.27 | 0.15 | 0.62 | 0.30 | 0.03 | 0.10 | 6.34 | 0.12 | 0.16 | 0.08 | 17.53 |
| 1 | 12 | Ask | 3 | 21 | 0.12 | 0.30 | 0.41 | 0.32 | 0.00 | 0.83 | 0.22 | 0.00 | 0.27 | 0.05 | 0.00 | 0.00 | 0.01 | 0.09 | 6.37 | 0.12 | 0.14 | 0.08 | 10.38 |
| 1 | 12 | Ask | 3 | 28 | 0.25 | 0.36 | 0.14 | 0.00 | 0.00 | 1.47 | 0.05 | 0.00 | 0.27 | 0.04 | 0.15 | 0.11 | 0.01 | 0.08 | 6.35 | 0.12 | 0.14 | 0.08 | 11.44 |
| 2 | 13 | Fes | 2 | 21 |  |  |  |  |  |  |  |  |  |  |  |  |  |  |  |  |  |  |  |
| 2 | 13 | Fes | 2 | 28 |  |  |  |  |  |  |  |  |  |  |  |  |  |  |  |  |  |  |  |
| 2 | 16 | Ask | 4 | 21 | 0.38 | 0.46 | 0.44 | 0.06 | 0.00 | 1.51 | 0.50 | 0.00 | 0.27 | 0.07 | 0.69 | 0.35 | 0.01 | 0.08 | 6.36 | 0.12 | 0.14 | 0.08 | 13.41 |
| 2 | 16 | Ask | 4 | 28 | 0.13 | 0.14 | 0.45 | 0.00 | 0.00 | 0.50 | 0.00 | 0.00 | 0.28 | 0.14 | 0.00 | 0.00 | 0.02 | 0.11 | 6.35 | 0.12 | 0.14 | 0.08 | 9.15 |
| 2 | 18 | Ask | 2 | 21 | 1.46 | 0.77 | 0.92 | 0.26 | 0.00 | 1.27 | 0.89 | 0.00 | 0.27 | 0.01 | 0.91 | 0.12 | 0.01 | 0.08 | 6.36 | 0.12 | 0.14 | 0.08 | 15.25 |
| 2 | 18 | Ask | 2 | 28 | 1.31 | 0.43 | 0.67 | 0.38 | 0.00 | 1.45 | 0.62 | 0.00 | 0.27 | 0.00 | 0.59 | 0.15 | 0.00 | 0.07 | 6.36 | 0.12 | 0.14 | 0.08 | 14.41 |
| 2 | 19 | Fes | 4 | 21 |  |  |  |  |  |  |  |  |  |  |  |  |  |  |  |  |  |  |  |
| 2 | 19 | Fes | 4 | 28 |  |  |  |  |  |  |  |  |  |  |  |  |  |  |  |  |  |  |  |
| **B** | **P** | **Var** | **N** | **DPA** | **Asn** | **Gln** | **Asp** | **Glu** | **Thr** | **Ala** | **Gly** | **Val** | **BABA** | **Ile** | **Ser** | **Pro** | **Met** | **Phe** | **Tyr** | **Orn** | **His** | **Trp** | **Total** |
| 2 | 20 | Ask | 1 | 21 | 0.72 | 1.81 | 0.26 | 0.49 | 0.00 | 2.03 | 0.00 | 0.01 | 0.00 | 0.00 | 0.30 | 0.50 | 0.03 | 0.09 | 6.37 | 0.12 | 0.16 | 0.08 | 15.49 |
| 2 | 20 | Ask | 1 | 28 | 0.55 | 0.70 | 0.05 | 0.00 | 0.00 | 1.07 | 0.03 | 0.00 | 0.00 | 0.01 | 0.12 | 0.22 | 0.01 | 0.07 | 6.38 | 0.12 | 0.14 | 0.08 | 10.87 |
| 2 | 21 | Fes | 1 | 21 | 0.93 | 0.98 | 0.53 | 0.17 | 0.00 | 0.86 | 0.02 | 0.00 | 0.28 | 0.05 | 0.15 | 0.14 | 0.01 | 0.08 | 6.37 | 0.12 | 0.15 | 0.08 | 12.01 |
| 2 | 21 | Fes | 1 | 28 | 1.31 | 1.59 | 0.54 | 0.39 | 0.00 | 1.98 | 0.17 | 0.00 | 0.00 | 0.07 | 0.67 | 0.36 | 0.02 | 0.09 | 6.36 | 0.12 | 0.15 | 0.08 | 16.38 |
| 2 | 22 | Fes | 3 | 21 | 0.11 | 0.33 | 0.74 | 0.02 | 0.00 | 0.89 | 0.19 | 0.00 | 0.28 | 0.10 | 0.56 | 0.13 | 0.01 | 0.09 | 6.36 | 0.12 | 0.14 | 0.08 | 11.31 |
| 2 | 22 | Fes | 3 | 28 | 0.35 | 0.43 | 2.22 | 0.00 | 0.00 | 0.19 | 0.00 | 0.23 | 0.27 | 0.36 | 0.00 | 0.31 | 0.02 | 0.13 | 6.32 | 0.12 | 0.15 | 0.09 | 11.57 |
| 2 | 24 | Ask | 3 | 21 | 0.15 | 0.35 | 0.25 | 0.00 | 0.00 | 1.34 | 0.10 | 0.00 | 0.28 | 0.04 | 0.39 | 0.00 | 0.02 | 0.08 | 6.36 | 0.12 | 0.14 | 0.08 | 11.36 |
| 2 | 24 | Ask | 3 | 28 | 0.15 | 0.46 | 0.42 | 0.71 | 0.00 | 1.13 | 0.27 | 0.00 | 0.28 | 0.04 | 0.36 | 0.04 | 0.02 | 0.08 | 6.38 | 0.12 | 0.14 | 0.08 | 12.09 |
| 3 | 26 | Fes | 2 | 21 | 1.10 | 0.76 | 0.68 | 0.07 | 0.00 | 1.98 | 0.53 | 0.03 | 0.00 | 0.09 | 1.49 | 0.32 | 0.02 | 0.09 | 6.36 | 0.12 | 0.16 | 0.08 | 16.36 |
| 3 | 26 | Fes | 2 | 28 | 1.13 | 0.43 | 0.34 | 0.23 | 0.00 | 0.97 | 0.57 | 0.00 | 0.28 | 0.00 | 0.53 | 0.17 | 0.01 | 0.07 | 6.36 | 0.12 | 0.14 | 0.08 | 12.62 |
| 3 | 27 | Fes | 1 | 21 | 0.99 | 1.19 | 0.72 | 0.52 | 0.00 | 1.76 | 0.25 | 0.03 | 0.00 | 0.16 | 0.82 | 0.31 | 0.03 | 0.10 | 6.36 | 0.12 | 0.15 | 0.08 | 15.79 |
| 3 | 27 | Fes | 1 | 28 | 0.95 | 1.00 | 0.35 | 0.01 | 0.00 | 1.61 | 0.06 | 0.00 | 0.27 | 0.05 | 0.32 | 0.11 | 0.02 | 0.08 | 6.38 | 0.12 | 0.14 | 0.08 | 13.56 |
| 3 | 28 | Ask | 4 | 21 | 0.32 | 0.38 | 0.87 | 0.04 | 0.00 | 1.00 | 0.58 | 0.00 | 0.28 | 0.13 | 1.07 | 0.25 | 0.02 | 0.10 | 6.36 | 0.12 | 0.14 | 0.08 | 13.04 |
| 3 | 28 | Ask | 4 | 28 | 0.47 | 0.48 | 0.18 | 0.07 | 0.00 | 1.88 | 0.22 | 0.01 | 0.27 | 0.07 | 1.17 | 0.25 | 0.02 | 0.09 | 6.37 | 0.12 | 0.15 | 0.08 | 14.24 |
| 3 | 29 | Ask | 3 | 21 | 0.32 | 0.35 | 0.35 | 0.00 | 0.00 | 1.13 | 0.34 | 0.02 | 0.27 | 0.21 | 0.74 | 0.15 | 0.03 | 0.10 | 6.34 | 0.12 | 0.15 | 0.08 | 12.18 |
| 3 | 29 | Ask | 3 | 28 | 0.39 | 0.33 | 0.21 | 0.00 | 0.00 | 1.79 | 0.15 | 0.02 | 0.27 | 0.12 | 0.78 | 0.22 | 0.03 | 0.10 | 6.37 | 0.12 | 0.14 | 0.08 | 13.35 |
| 3 | 31 | Ask | 2 | 21 | 0.77 | 0.89 | 0.68 | 0.72 | 0.00 | 2.18 | 0.90 | 0.04 | 0.28 | 0.12 | 0.81 | 0.36 | 0.02 | 0.10 | 6.37 | 0.12 | 0.16 | 0.08 | 17.30 |
| 3 | 31 | Ask | 2 | 28 | 0.51 | 0.38 | 0.56 | 0.00 | 0.00 | 0.54 | 0.34 | 0.00 | 0.27 | 0.00 | 0.42 | 0.10 | 0.01 | 0.08 | 6.38 | 0.12 | 0.14 | 0.08 | 10.59 |
| 3 | 33 | Ask | 1 | 21 | 0.86 | 1.36 | 1.21 | 0.35 | 0.00 | 1.16 | 0.12 | 0.00 | 0.28 | 0.07 | 0.69 | 0.18 | 0.02 | 0.08 | 6.38 | 0.12 | 0.14 | 0.08 | 14.57 |
| 3 | 33 | Ask | 1 | 28 | 0.28 | 0.47 | 0.27 | 0.00 | 0.00 | 0.73 | 0.00 | 0.00 | 0.28 | 0.00 | 0.16 | 0.10 | 0.01 | 0.07 | 6.39 | 0.12 | 0.14 | 0.08 | 10.01 |
| 3 | 34 | Fes | 4 | 21 |  |  |  |  |  |  |  |  |  |  |  |  |  |  |  |  |  |  |  |
| 3 | 34 | Fes | 4 | 28 | 0.47 | 0.62 | 0.00 | 0.00 | 0.00 | 2.05 | 0.00 | 0.01 | 0.00 | 0.12 | 0.56 | 1.04 | 0.03 | 0.10 | 6.37 | 0.12 | 0.15 | 0.08 | 14.30 |
| 3 | 35 | Fes | 3 | 21 |  |  |  |  |  |  |  |  |  |  |  |  |  |  |  |  |  |  |  |
| 3 | 35 | Fes | 3 | 28 | 0.69 | 1.44 | 0.23 | 0.06 | 0.00 | 4.18 | 0.11 | 0.08 | 0.00 | 0.11 | 0.63 | 1.41 | 0.04 | 0.09 | 6.38 | 0.12 | 0.15 | 0.08 | 20.92 |

**Table S5.** Gene expression data for rye cv. Askari grain from plants grown under different sulfur treatments, with nitrogen supplied (N^+^). Tissue was harvested at 21 dpa.

**a)** Normalised relative quantities (NRQs) of candidate gene transcripts

| **Target** | **Normalised relative quantity of transcript** | | | | | |
| --- | --- | --- | --- | --- | --- | --- |
|  | **Block 1** | | **Block 2** | | **Block 3** | |
|  | **S^+^** | **S^-^** | **S^+^** | **S^-^** | **S^+^** | **S^-^** |
| ***ScASN1*** | 1.71E+16 | 2.39E+17 | 7.3E+15 | 3.64E+16 | 3.09E+14 | 6.76E+14 |
| ***ScSnRK1*** | 4.83E+18 | 3.27E+18 | 5.14E+15 | 5.28E+17 | 7.9E+15 | 5.21E+15 |
| ***ScASK1*** | 1.02E+17 | 4.53E+16 | 1.00E+16 | 1.30E+16 | 1.48E+14 | 9.60E+13 |
| ***ScASP1*** | 6.10E+16 |  | 8.53E+16 | 7.77E+15 | 1.60E+14 | 3.15E+14 |
| ***ScGCN2*** | 2.23E+16 | 1.04E+16 | 3.72E+15 | 3.88E+15 | 3.82E+13 | 2.96E+13 |
| ***ScGS1-1*** | 2.54E+17 | 3.36E+17 | 4.88E+16 | 6.40E+16 | 1.81E+15 | 1.51E+15 |

**b)** Log transformed NRQs used for statistical analysis; p-values from statistical tests of differences between means are shown.

| **Target** | **Log_2_ (1/NRQ)** | | | | | | **p values** | |
| --- | --- | --- | --- | --- | --- | --- | --- | --- |
|  | **Block 1** | | **Block 2** | | **Block 3** | |  |  |
|  | **S^+^** | **S^-^** | **S^+^** | **S^-^** | **S^+^** | **S^-^** | **t-test*** | **ANOVA**** |
| ***ScASN1*** | -53.93 | -57.73 | -52.70 | -55.02 | -48.13 | -49.26 | 0.473 | 0.089 |
| ***ScSnRK1*** | -62.07 | -61.50 | -52.19 | -58.87 | -52.81 | -52.21 | 0.686 | 0.527 |
| ***ScASK1*** | -56.50 | -55.33 | -53.15 | -53.53 | -47.08 | -46.45 | 0.909 | 0.408 |
| ***ScASP1*** | -55.76 |  | -56.24 | -52.79 | -47.18 | -48.16 | 0.579 | 0.618 |
| ***ScGCN2*** | -54.31 | -53.20 | -51.73 | -51.78 | -45.12 | -44.75 | 0.907 | 0.301 |
| ***ScGS1-1*** | -57.82 | -58.22 | -55.44 | -55.83 | -50.68 | -50.42 | 0.957 | 0.499 |

*t-tests were performed to test for significant differences between treatments.

**Analysis of variance was performed to test (F-tests) for significant differences between treatments and also takes into account the block structure of the experiment.

**Table S6.** Gene expression data for rye cv. Askari grain from plants grown under different sulfur treatments, with nitrogen supplied (N^+^). Tissue was harvested at 28 dpa.

**a)** Normalised relative quantities (NRQs) of candidate gene transcripts

|  | **Normalised relative quantity of transcript** | | | | | |
| --- | --- | --- | --- | --- | --- | --- |
|  | **Block 1** | | **Block 2** | | **Block 3** | |
|  | **S^+^** | **S^-^** | **S^+^** | **S^-^** | **S^+^** | **S^-^** |
| ***ScASN1*** | 2.97E+15 | 2.27E+17 | 1.76E+14 | 1.61E+17 | 3.87E+16 | 1.86E+16 |
| ***ScSnRK1*** | 4.01E+16 | 5.05E+16 | 8.04E+15 | 4.21E+16 | 8.40E+16 | 1.39E+17 |
| ***ScASK1*** | 7.78E+15 | 3.50E+15 | 1.52E+15 | 8.97E+15 | 3.76E+16 | 6.32E+15 |
| ***ScGS1-1*** | 2.96E+16 | 9.53E+16 | 6.98E+15 | 1.14E+17 | 1.47E+17 | 4.12E+16 |

**b)** Log transformed NRQs used for statistical analysis; p-values from statistical tests of differences between means are shown

| **Target** | **Log_2_ (1/NRQ)** | | | | | | **p values** | |
| --- | --- | --- | --- | --- | --- | --- | --- | --- |
|  | **Block 1** | | **Block 2** | | **Block 3** | | **t-test*** | **ANOVA**** |
|  | **S^+^** | **S^-^** | **S^+^** | **S^-^** | **S^+^** | **S^-^** |  |  |
| ***ScASN1*** | -51.40 | -57.66 | -47.32 | -57.16 | -55.10 | -54.04 | 0.117 | 0.259 |
| ***ScSnRK1*** | -55.16 | -55.49 | -52.84 | -55.22 | -56.22 | -56.95 | 0.368 | 0.210 |
| ***ScASK1*** | -52.79 | -51.63 | -50.44 | -52.99 | -55.06 | -52.49 | 0.794 | 0.823 |
| ***ScGS1-1*** | -54.72 | -56.40 | -52.63 | -56.67 | -57.03 | -55.19 | 0.670 | 0.765 |

*t-tests were performed to test for significant differences between treatments.

**Analysis of variance was performed to test (F-tests) for significant differences between treatments and also takes into account the block structure of the experiment.

**Table S7.** Gene expression data for rye cv. Askari flag leaf from plants grown under different sulfur treatments, with nitrogen supplied (N^+^). Tissue was harvested at 28 dpa.

a) Normalised relative quantities (NRQs) of candidate gene transcripts

| **Target** | **Normalised relative quantity of transcript** | | | | | |
| --- | --- | --- | --- | --- | --- | --- |
|  | **Block 1** | | **Block 2** | | **Block 3** | |
|  | **S^+^** | **S^-^** | **S^+^** | **S^-^** | **S^+^** | **S^-^** |
| ***ScASN1*** | 2.34E+17 |  | 1.57E+17 | 5.13E+20 | 6.82E+17 | 1.33E+18 |
| ***ScASK1*** | 3.25E+18 |  | 1.30E+18 | 2.10E+19 | 3.53E+18 | 2.00E+17 |
| ***ScGS1-1*** | 1.95E+19 |  | 7.42E+18 | 3.08E+20 | 6.58E+19 | 3.57E+18 |

**b)** Log transformed NRQs used for statistical analysis; p-values from statistical tests of differences between means are shown.

| **Target** | **Log 2 (1/NRQ)** | | | | | | **p values** | |
| --- | --- | --- | --- | --- | --- | --- | --- | --- |
|  | **Block 1** | | **Block 2** | | **Block 3** | |  |  |
|  | **S^+^** | **S^-^** | **S^+^** | **S^-^** | **S^+^** | **S^-^** | **t-test*** | **ANOVA**** |
| ***ScASN1*** | -57.70 |  | -57.12 | -68.80 | -59.24 | -60.21 | 0.145 | 0.385 |
| ***ScASK1*** | -61.50 |  | -60.17 | -64.19 | -61.62 | -57.47 | 0.924 | 0.987 |
| ***ScGS1-1*** | -64.08 |  | -62.69 | -68.06 | -65.84 | -61.63 | 0.825 | 0.906 |

*t-tests were performed to test for significant differences between treatments.

**Analysis of variance was performed to test (F-tests) for significant differences between treatments and also takes into account the block structure of the experiment.

**Table S8** Gene expression data for rye cv. Askari flag leaf from plants grown under different nitrogen treatments, with sulfur supplied (S^+^). Tissue was harvested at 28 dpa.

**a)** Normalised relative quantities (NRQs) of candidate gene transcripts

| **Target** | **Normalised relative quantity of transcript** | | | | | |
| --- | --- | --- | --- | --- | --- | --- |
|  | **Block 1** | | **Block 2** | | **Block 3** | |
|  | **N^+^** | **N^-^** | **N^+^** | **N^-^** | **N^+^** | **N^-^** |
| ***ScASN1*** | 3.64E+17 | 1.08E+16 | 1.51E+17 | 1.76E+18 | 7.69E+17 | 4.38E+16 |
| ***ScSnRK1*** |  | 1.09E+17 | 2.14E+17 | 4.47E+18 | 7.62E+17 | 7.68E+16 |
| ***ScASK1*** | 2.19E+18 | 3.99E+17 | 1.19E+18 | 1.57E+19 | 2.12E+20 | 7.15E+17 |
| ***ScASP1*** | 2.60E+17 | 2.65E+16 | 1.26E+17 | 4.29E+18 |  | 8.95E+16 |
| ***ScGS1-1*** | 4.65E+18 | 1.47E+18 | 2.26E+18 | 3.99E+19 | 1.00E+19 | 7.62E+18 |

**b)** Log transformed NRQs used for statistical analysis; p-values from statistical tests of differences between means are shown.

| **Target** | **Log 2 (1/NRQ)** | | | | | | **p values** | |
| --- | --- | --- | --- | --- | --- | --- | --- | --- |
|  | **Block 1** | | **Block 2** | | **Block 3** | |  |  |
|  | **N^+^** | **N^-^** | **N^+^** | **N^-^** | **N^+^** | **N^-^** | **t-test*** | **ANOVA**** |
| ***ScASN1*** | -58.34 | -53.27 | -57.06 | -60.61 | -59.42 | -55.28 | 0.457 | 0.561 |
| ***ScSnRK1*** |  | -56.60 | -57.57 | -61.96 | -59.40 | -56.09 | 0.921 | 0.893 |
| ***ScASK1*** | -60.93 | -58.47 | -60.05 | -63.77 | -67.52 | -59.31 | 0.924 | 0.570 |
| ***ScASP1*** | -57.85 | -54.56 | -56.80 | -61.90 |  | -56.31 | 0.933 | 0.837 |
| ***ScGS1-1*** | -62.01 | -60.35 | -60.97 | -65.11 | -63.12 | -62.73 | 0.669 | 0.731 |

*t-tests were performed to test for significant differences between treatments.

**Analysis of variance was performed to test (F-tests) for significant differences between treatments and also takes into account the block structure of the experiment.

**Table S9** Gene expression data for rye cv. Askari grain from plants grown under nitrogen and sulfur sufficiency (N^+^S^+^). Tissue was harvested at 28 and 21 dpa.

**a)** Normalised relative quantities (NRQs) of candidate gene transcripts

| **Target** | **Normalised relative quantity of transcript** | | | | | |
| --- | --- | --- | --- | --- | --- | --- |
|  | **Block 1** | | **Block 2** | | **Block 3** | |
|  | **28 dpa** | **21 dpa** | **28 dpa** | **21 dpa** | **28 dpa** | **21 dpa** |
| ***ScASN1*** | 5.30E+15 | 3.84E+15 | 4.06E+14 | 7.49E+15 | 1.64E+16 | 2.70E+14 |
| ***ScSnRK1*** | 5.72E+16 | 4.79E+17 | 9.33E+15 | 2.77E+17 | 3.56E+16 | 7.41E+15 |
| ***ScASK1*** | 9.09E+15 | 3.85E+16 | 8.24E+14 | 1.83E+16 | 3.24E+16 | 3.74E+14 |
| ***ScGS1-1*** | 1.93E+16 | 7.21E+16 | 3.18E+15 | 8.07E+16 | 1.22E+17 | 1.65E+15 |

**b)** Log transformed NRQs used for statistical analysis; p-values from statistical tests of differences between means are shown.

| **Target** | **Log_2_ (1/NRQ)** | | | | | | **p values** | |
| --- | --- | --- | --- | --- | --- | --- | --- | --- |
|  | **Block 1** | | **Block 2** | | **Block 3** | |  |  |
|  | **28 dpa** | **21 dpa** | **28 dpa** | **21 dpa** | **28 dpa** | **21 dpa** | **t-test*** | **ANOVA**** |
| ***ScASN1*** | -52.24 | -51.77 | -48.53 | -52.73 | -53.87 | -47.94 | 0.752 | 0.827 |
| ***ScSnRK1*** | -55.67 | -58.73 | -53.05 | -57.94 | -54.98 | -52.72 | 0.406 | 0.470 |
| ***ScASK1*** | -53.01 | -55.10 | -49.55 | -54.02 | -54.85 | -48.41 | 0.988 | 0.992 |
| ***ScGS1-1*** | -54.10 | -56.00 | -51.50 | -56.16 | -56.76 | -50.55 | 0.963 | 0.974 |

*t-tests were performed to test for significant differences between treatments.

**Analysis of variance was performed to test (F-tests) for significant differences between treatments and also takes into account the block structure of the experiment.
